# Homeobox Gene Duplication and Divergence in Arachnids

**DOI:** 10.1093/molbev/msy125

**Published:** 2018-06-19

**Authors:** Daniel J Leite, Luís Baudouin-Gonzalez, Sawa Iwasaki-Yokozawa, Jesus Lozano-Fernandez, Natascha Turetzek, Yasuko Akiyama-Oda, Nikola-Michael Prpic, Davide Pisani, Hiroki Oda, Prashant P Sharma, Alistair P McGregor

**Affiliations:** 1 Department of Biological and Medical Sciences, Oxford Brookes University, Oxford, United Kingdom; 2 JT Biohistory Research Hall, Takatsuki, Osaka, Japan; 3 School of Earth Sciences, University of Bristol, Life Sciences Building, Bristol, United Kingdom; 4 School of Biological Sciences, University of Bristol, Life Sciences Building, Bristol, United Kingdom; 5 Department of Cellular Neurobiology, Johann-Friedrich-Blumenbach Institute for Zoology and Anthropology, Georg-August-University, Göttingen, Germany; 6 Microbiology and Infection Control, Osaka Medical College, Takatsuki, Osaka, Japan; 7 Department of Biological Sciences, Graduate School of Science, Osaka University, Osaka, Japan; 8 Department of Integrative Biology, University of Wisconsin-Madison, Madison, WI, USA

**Keywords:** homeobox genes, development, gene duplication

## Abstract

Homeobox genes are key toolkit genes that regulate the development of metazoans and changes in their regulation and copy number have contributed to the evolution of phenotypic diversity. We recently identified a whole genome duplication (WGD) event that occurred in an ancestor of spiders and scorpions (Arachnopulmonata), and that many homeobox genes, including two Hox clusters, appear to have been retained in arachnopulmonates. To better understand the consequences of this ancient WGD and the evolution of arachnid homeobox genes, we have characterized and compared the homeobox repertoires in a range of arachnids. We found that many families and clusters of these genes are duplicated in all studied arachnopulmonates (*Parasteatoda tepidariorum*, *Pholcus phalangioides, Centruroides sculpturatus*, and *Mesobuthus martensii*) compared with nonarachnopulmonate arachnids (*Phalangium opilio*, *Neobisium carcinoides, Hesperochernes* sp., and *Ixodes scapularis*). To assess divergence in the roles of homeobox ohnologs, we analyzed the expression of *P. tepidariorum* homeobox genes during embryogenesis and found pervasive changes in the level and timing of their expression. Furthermore, we compared the spatial expression of a subset of *P. tepidariorum* ohnologs with their single copy orthologs in *P. opilio* embryos. We found evidence for likely subfunctionlization and neofunctionalization of these genes in the spider. Overall our results show a high level of retention of homeobox genes in spiders and scorpions post-WGD, which is likely to have made a major contribution to their developmental evolution and diversification through pervasive subfunctionlization and neofunctionalization, and paralleling the outcomes of WGD in vertebrates.

## Introduction

Developmental programs precisely orchestrate proliferation and differentiation to build multicellular organisms. Many of the key regulatory factors and pathways utilized in development are conserved between species like the Wnt and Delta/Notch signaling pathways and transcription factors (TF) such as those encoded by the homeobox genes (Reviewed by Carroll *et al.* 2005; Rokas 2008). Many studies in recent decades have shown that changes in the expression and copy number of these tool kit genes can lead to the evolution of phenotypic differences among species (Averof and Patel 1997; Stern 1998; Ronshaugen *et al.* 2002; Carroll *et al.* 2005; Liubicich *et al.* 2009; Werner *et al.* 2010; Guerreiro *et al.* 2013; Koshikawa *et al.* 2015; Kvon *et al.* 2016). Therefore, understanding the evolution of these genes can provide important insights into the development and evolution of metazoans.

The homeobox genes encode a large superclass of TFs (Garcia-Fernandez 2005; Hoegg and Meyer 2005; Pascual-Anaya *et al.* 2012; Holland 2015; Ferrier 2016). They are characterized by encoding a homeodomain, which is usually 60 amino acids in length and folds to form a structure with three α-helices and an N-terminal domain (Ortiz-Lombardia *et al.* 2017). The third α-helix and N-terminal domain confer the specificity to the binding of the homeodomain to the major and minor groove of the DNA double helix, respectively (Hanes and Brent 1991; Chu *et al.* 2012; Ortiz-Lombardia *et al.* 2017). This conservation of sequence facilitates the characterization of many homeobox genes based solely on their homeodomain sequence (Holland *et al.* 2007), although there are also a variety of other DNA binding domains found in metazoan homeobox genes, which provide additional identification characteristics and biological functions (Burglin and Affolter 2016).

During the evolution of metazoans the expansion of homeobox gene number via duplication has been associated with multicellularity and the increase in morphological complexity (Garcia-Fernandez 2005; Hoegg and Meyer 2005; Pascual-Anaya *et al.* 2012; Holland 2015). The initial multiplication and divergence of proto-homeobox genes started early in evolution and created several classes of homeobox genes (Pascual-Anaya *et al.* 2012; Ferrier 2016). In the urbilaterian, the homeobox genes are hypothesized to have formed a large “Giga-homeobox” cluster, containing several homeobox families (Ferrier 2016). In metazoans, this Giga-cluster also included the metazoan specific ANTP class of homeobox genes (Ferrier 2016). Subsequent tandem duplications of each of the different classes generated clusters of similar homeobox class genes such as the ParaHox, SuperHox, SINE/Six, TALE/Irx, PRD/HRO clusters (Ferrier 2016). These clusters were then fragmented in the genome of the bilaterian ancestor, and have been subject to lineage specific retention, loss, and further duplication during bilaterian evolution (Ferrier 2016).

We recently found that in arachnids there had been a whole genome duplication (WGD) in a common ancestor of arachnopulmonates (spiders, scorpions, and Pedipalpi [Uropygi and Amblypygi]; Sharma, Kaluziak, *et al.* 2014; Schwager *et al.* 2017). Like the independent WGDs in vertebrates, after this event many duplicated homeobox genes have been retained in spiders and scorpions, including two clusters of Hox genes (Lynch *et al.* 2006; Putnam *et al.* 2008; Cao *et al.* 2013; Sharma, Schwager, *et al.* 2014; Di *et al.* 2015; Qu *et al.* 2015; Sharma et al. 2015; Schwager *et al.* 2017). Furthermore, divergence in the expression of ohnologs in spiders, including the Hox genes, suggests there has been neofunctionalization and subfunctionalization of many of these genes since the WGD (Pechmann *et al.* 2015; Turetzek *et al.* 2016, 2017; Schwager *et al.* 2017).

Here, we systematically compare the repertoires of homeobox genes between the arachnopulmonates with an ancestral WGD, the spiders *Parasteatoda tepidariorum* and *Pholcus phalangioides*, and the scorpions *Centruroides sculpturatus* and *Mesobuthus martensii* (Di *et al.* 2015), with arachnids that have no evidence for an ancestral WGD, the harvestman *Phalangium opilio*, the pseudoscorpions *Neobisium carcinoides* and *Hesperochernes* sp., and the tick *Ixodes scapularis*, as well as several mandibulate arthropods. We find pervasive duplication and retention of homeobox genes in arachnopulmonates, and synteny analysis of homeobox genes in *P. tepidariorum* also revealed several more duplicated ancient homeobox clusters (Ferrier 2016), in addition to the Hox clusters. To explore the fate and role of these duplicated genes further we compared the expression profiles of ohnologs during spider embryogenesis and found striking differences in their levels and temporal expression. Furthermore, comparison of the spatial expression of duplicated homeobox genes between *P. tepidariorum* and their single copy homologues in *P. opilio* suggests that there has been extensive neofunctionalization and subfunctionalization in embryogenesis during arachnopulmonate evolution. Taken together, our work shows that WGD greatly expanded the repertoire of homeobox genes in arachnopulmonates and that this contributed to diversification in their developmental gene regulatory networks and may have contributed to evolutionary innovations in these animals as has been postulated in other animal lineages (Van de Peer *et al.* 2009; Huminiecki and Conant 2012).

## Results

### Comparison of Homeobox Gene Families in Arachnids and Other Arthropods

**Fig. 1.**
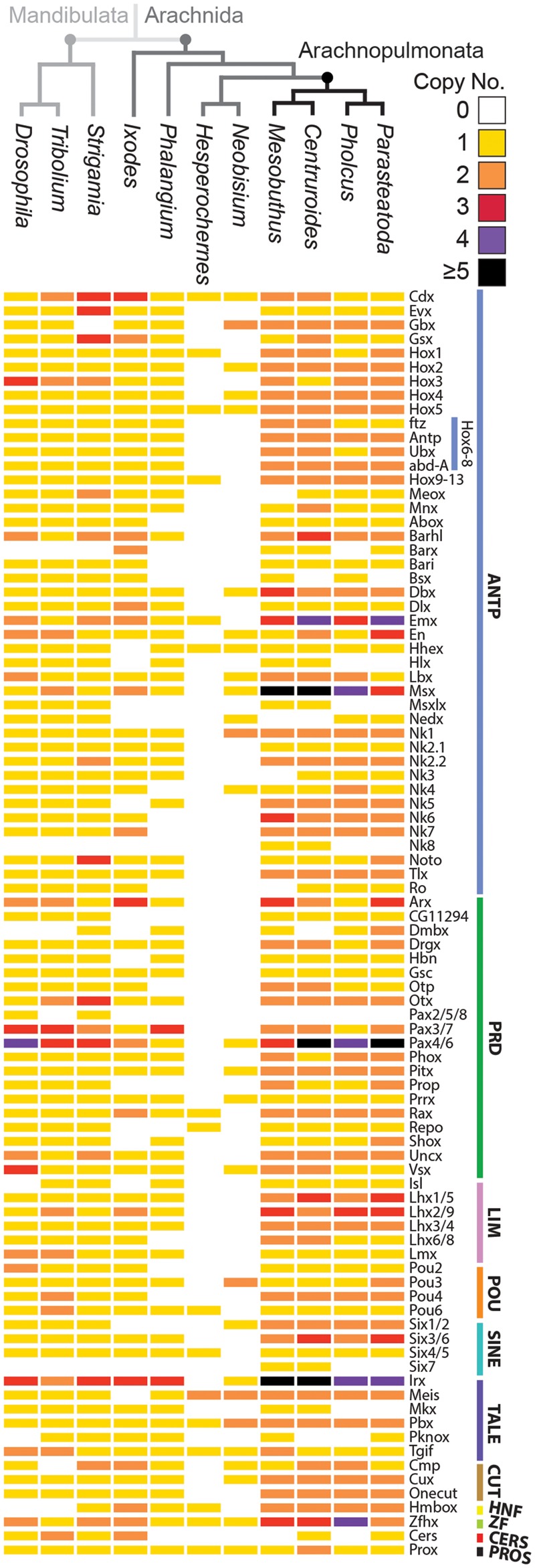
Comparison of homeobox repertoires in arthropods reveals pervasive duplication in arachnopulmonates. The copy number of homeobox families is generally greater in arachnopulmonates compared with other arthropods across all classes, except Cers and Pros. Homeobox genes are classified based on Holland *et al.* (2007) and the number of paralogs in each family is color coded. The Hox6-8 family has been broken down further to show the specific copy numbers of *ftz*, *Antp*, *Ubx*, and *abdA*.

To systematically identify homeobox repertoires we searched for the characteristic homeodomain sequence in a range of available and new arachnid transcriptomes. In a transcriptome of the spider *P. phalangioides* (Turetzek *et al.*, in preparation), we identified 78 homeobox families (fig. 1 and supplementary file 1, Supplementary Material online), which is similar to the 80 families identified previously in the spider *P. tepidariorum* (Schwager *et al.* 2017) and to the 82 families found in the scorpions *C. sculpturatus* and *M. martensii* (Di *et al.* 2015; Schwager *et al.* 2017).

For lineages that were thought not to have an ancestral WGD, we surveyed existing transcriptomes from the tick *I. scapularis*, the harvestman *P. opilio*, and the pseudoscorpion *Hesperochernes* sp., as well as sequencing a transcriptome for another pseudoscorpion *N. carcinoides*. The number of homeobox families found in *I. scapularis* (70) (fig. 1 and supplementary file 1, Supplementary Material online) was comparable to arachnopulmonates and mandibulates (*S. maritima*—83; *A. mellifera*—77; *T. castaneum*—80; *D. melanogaster*—80) (Zhong *et al.* 2008; Zhong and Holland 2011; Chipman *et al.* 2014). However, we only managed to recover genes from 65 families in *P. opilio* and just 27 and 16 families in *N. carcinoides* and *Hesperochernes* sp., respectively, which likely represent only a subset of families present in these arachnids (fig. 1 and supplementary file 1, Supplementary Material online).

The assignment of homeobox genes into families was verified using a maximum likelihood tree constructed using the homeodomain sequences (supplementary fig. 1, Supplementary Material online). This analysis provided good support for the annotation of each homeodomain to a homeobox gene family, as families were generally monophyletic and had >70% bootstrap support. The general topology of the tree also grouped the homeobox classes together consistent with Holland *et al.* (2007).

Comparisons of the repertoires of homeobox families between these species suggest particular patterns of retention and loss of homeobox families in arthropod lineages (fig. 1). Overall, excluding the harvestman and pseudoscorpion data due to incompleteness, 60 of the known 87 homeobox families were present in all species surveyed, indicating a reasonable retention of most families.

Families that were present in vertebrates, arachnids and the myriapod, but absent in insects were the HNF and Dmbx families (Zhong *et al.* 2008; Zhong and Holland 2011; Chipman *et al.* 2014). Another family that was present in vertebrates and arachnids but missing from the mandibulates surveyed was the Barx family (Zhong *et al.* 2008; Zhong and Holland 2011; Chipman *et al.* 2014). The only family not present in arachnids but present in mandibulates and vertebrates was the Pax2/5/8 family.

There were also some retention/loss differences among arachnid species. While Nedx is present in spiders, it appears to have been lost in the scorpions and *I. scapularis*, although there is a single copy in the pseudoscorpion *N. carcinoides* (fig. 1 and supplementary file 1, Supplementary Material online). The Hlx, Msxlx, and Mkx families also appear to be missing from spiders but present in the scorpions and the mandibulates surveyed (fig. 1).

### Pervasive Duplication of Homeobox Genes in Arachnopulmonates

Although the number of homeobox families is fairly similar between arthropod species surveyed, except the harvestman and pseudoscorpions, the actual number of genes varied considerably between arachnopulmonates and nonarachnopulmonate arthropods. The spider *P. phalangioides* had a total of 132 homeobox genes (supplementary file 1, Supplementary Material online), which is comparable to the 145 in *P. tepidariorum* and the 156 found in the scorpions *C. sculpturatus* and *M. martensii* (Di *et al.* 2015; Schwager *et al.* 2017). In contrast, the nonarachnopulmonate species *I. scapularis*, *P. opilio*, *N. carcinoides*, and *Hesperochernes* sp. had 96, 69, 32, and 17 homeobox genes, respectively (supplementary file 1, Supplementary Material online). The most complete nonarachnopulmonate data set represented by *I. scapularis* compared well to the number of homeobox genes previously identified in *S. maritima* (113), *T. castaneum* (105), and *D. melanogaster* (104) (Zhong *et al.* 2008; Zhong and Holland 2011; Chipman *et al.* 2014).

We found that 58%, 50%, 59%, 57% of homeobox families in *P. tepidariorum*, *P. phalangioides*, *C. sculpturatus*, and *M. martensii* are duplicated, compared with 24% in the tick, 3% in the harvestman, 19% in the centipede, beetle, and fly. This shows that many more of the arachnopulmonate homeobox families are comprised of multiple genes copies compared with other arthropods. In total, 34 families are duplicated in all four arachnopulmonate species (fig. 1), which may indicate that these were duplicated in a single event and subsequently retained in the ancestor of the Araneae and Scorpiones lineages. 18 of these 34 families are not duplicated in any of the nonarachnopulmonate species surveyed. Furthermore, 38 families are duplicated in both spiders, whereas 46 families are duplicated in both scorpions (fig. 1).

The families in arachnopulmonates that contain more than two copies, such as Pax4/6 and Irx, are also duplicated in the mandibulate species surveyed. This perhaps suggests that these were duplicated in the arthropod ancestor and that further paralogs were generated in arachnopulmonates due to the WGD (fig. 1).

### Homeobox Gene Ohnologs and Tandem Duplicates in *P. tepidariorum*

It has already been shown that duplicated Hox clusters were retained after the ancestral WGD in arachnopulmonates (Schwager *et al.* 2017). Therefore, we next investigated if other homeobox gene clusters have also been retained. Of the 45 homeobox gene families that are duplicated in *P. tepidariorum*, 40 families are represented by paralogs that are located on different scaffolds, hereafter called dispersed paralogs. Some of these dispersed paralogs are present as duplicated clusters in the genome.

**Fig. 2.**
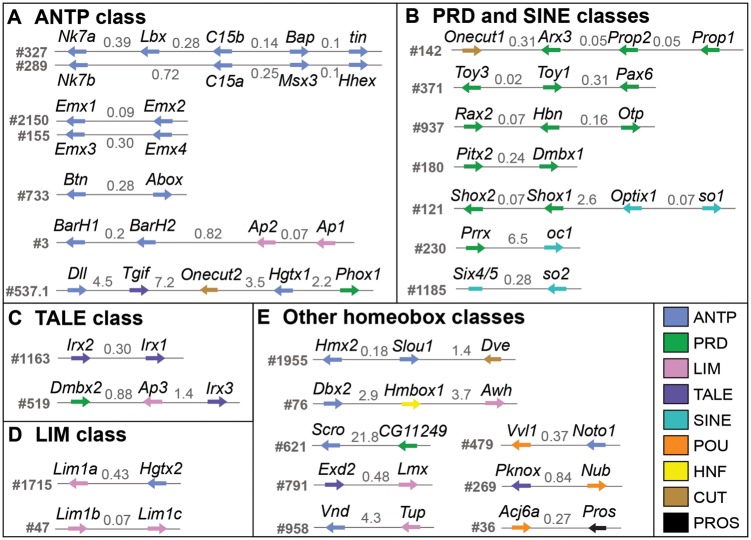
Homeobox gene clustering in the *Parasteatoda tepidariorum* genome. (*A*) Scaffolds containing at least two ANTP class genes. (*B*) Scaffolds containing PRD and SINE class gene clusters. (*C*) Scaffolds containing the Irx family of the TALE class. (*D*) Scaffolds with Lhx1/5 family of the LIM class. (*E*) Other scaffolds with at least two homeobox genes. All other homeobox genes were localized to individual scaffolds. The intergenic distances are indicated in Mb. *Parasteatoda tepidariorum* DoveTail assembly scaffold numbers are to the left of each cluster. Arrows depict the direction of transcription. Nonhomeobox genes are not shown.

One homeobox cluster that is present across protostomes and deuterostomes is the NK cluster (Garcia-Fernandez 2005; Ferrier 2016). In *P. tepidariorum*, we identified scaffolds that contained duplicated remnants of this cluster. There were two clusters that contained *Nk7* and *Tlx*/*C15* paralogs, which have the same transcriptional orientation on each scaffold (fig. 2*A*). Each of these clusters also contained other ANTP class genes that are usually found in the NK cluster (*Lbx*, *Bap*, *tin*, *Hhex*, and *Msx*). However, of these five genes only *Msx* is duplicated, though the other two *Msx* paralogs are not located in the NK clusters. This indicates differential retention/loss between these duplicate NK clusters in *P. tepidariorum*.

We also identified other clusters of homeobox genes that are duplicated and retained to various extent in *P. tepidariorum*. There is evidence for a duplication of the SINE/Six cluster on scaffolds #121 and #1185 (fig. 2*B*). This cluster, found in both protostomes and deuterostomes, is usually composed of three genes commonly arranged in the order *Optix*, *sine oculis* (*so*), and *Six4/5* (Ferrier 2016)*.* On both scaffolds there are *so* genes followed by one paralog of *Optix* on scaffold #121 and the single *Six4/5* gene on scaffold #1185. There are also other paralogs of *Optix* in *P. tepidariorum* but they are dispersed in the genome. We also identified clusters of ANTP, TALE, and LIM class genes. There are two scaffolds that each contained two tandem paralogs of *Emx* genes, and these clusters have maintained the same transcriptional orientation (fig. 2*A*). For the TALE class, two *Irx*/*mirr* paralogs were identified on one scaffold and a single copy of *Irx* was present on another scaffold along with *Dmbx2* and *Ap3* (fig. 2*C*). We also identified a scaffold containing two *Lhx1/5* paralogs and another with a single copy of *Lhx1/5* and one of the *Hgtx* paralogs (fig. 2*D*).

We also found eight homeobox families with tandemly duplicated paralogs: the BarH, Lhx5/9, Pax4/6, Prop, and Shox families as well as the aforementioned mentioned Emx, Irx, and Lhx1/5 families (fig. 2). These tandem duplicates were all found in the same transcriptional orientation apart from the Pax4/6 cluster. This means that of the retained duplicate homeobox families, 50% were found as dispersed paralogs, whereas only 6% have conclusively resulted from tandem duplications. Collectively this implies that there has been a greater contribution of WGD than tandem duplication to the expansion of arachnopulmonate homeobox repertoires.

### Expression of Homeobox Genes in a *P. tepidariorum* Embryogenesis

**Fig. 3.**
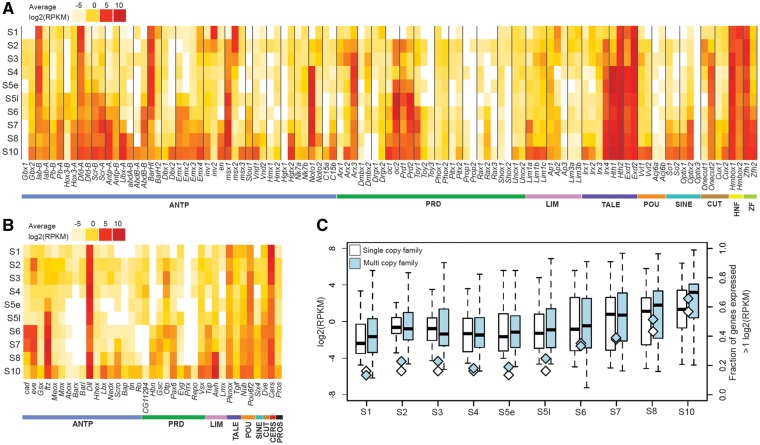
Expression of homeobox genes in *Parasteatoda tepidariorum* from S1 to S10. The transcriptome profile of *P. tepidariorum* AUGUSTUS gene models for (*A*) duplicated and (*B*) single copy Homeobox genes. (*C*) The average expression of all homeobox genes increases from S1 to S2, likely corresponding to onset of zygotic transcription (Pechmann *et al.* 2017). The numbers of families expressed >1 log2(RPKM) also increase from S1 to S2. The mean expression level is lower and fewer families are expressed around S4/S5e. After which the mean expression level and number of families continues to increase.

We next investigated the expression of homeobox genes in *P. tepidariorum* by quantifying their levels in RNA-Seq data covering the first ten stages of embryogenesis of this spider. All 145 annotated homeobox genes were found to be expressed in at least one of the ten embryonic stages assayed, with the exception of *Slou2* (fig. 3*A* and *B*).

There is an increase in the average expression of single copy and duplicated homeobox genes from S1 to S2 (fig. 3*C*). The number of homeobox genes expressed >1 log2(RPKM) also increases between these first two stages, especially in the case of the multicopy genes. This observation is likely to be explained by the onset of zygotic transcription at S2 (Pechmann *et al.* 2017). After S2 both the average expression level and number of genes expressed decreases to the lowest levels around early S5 after which the number of genes and the average expression also increases (fig. 3*C*).

Interestingly, one homeobox gene that is highly expressed at S1 was *Distal-less* (*Dll*) (fig. 3*B*). This is much earlier than previously reported at S5 (detected by ISH) and its roles in segment specification and limb development (Pechmann *et al.* 2011). Furthermore, expression of *Pt-cad* and *Pt-eve* was also earlier detected at S1 and then increased at S2, again earlier than previously detected using ISH (fig. 3*B*) (Schönauer *et al.* 2016). Therefore, it is possible that *Pt-Dll, Pt-cad*, *and Pt-eve* are maternally deposited in this spider and are involved in presently unknown functions during early embryogenesis.

### Expression Divergence of Duplicated *P. tepidariorum* Homeobox Genes in the Embryonic Transcriptome

To assess the divergence in the expression of duplicated *P. tepidariorum* homeobox genes, the RNA-Seq profiling was then analyzed to compare the expression levels of dispersed and tandem paralogs during embryogenesis in this spider (fig. 3*A*).

The spatial and temporal expression of Hox paralogs in *P. tepidariorum* was previously analyzed using ISH and showed that Hox genes from both clusters are expressed in the classical collinear fashion across the AP axis (Schwager *et al.* 2017). Interestingly, both the previous ISHs and our RNA-Seq profiling reveal that one paralog of each Hox gene is always expressed earlier than the other, except for the *Pt-abdA* paralogs (Schwager *et al.* 2017). Overall, the timing of Hox expression in the RNA-Seq data matches well with onset of expression detected by ISH (fig. 3*A*). However, both *Pt-lab-A* and *Pt-Dfd-A* were highly expressed from S1 onward, indicating earlier expression than detected by ISH (Pechmann *et al.* 2015; Schwager *et al.* 2017). These results are consistent with previous findings that *P. tepidariorum* Hox paralogs have probably been subject to subfunctionalization and/or neofunctionalization (Pechmann *et al.* 2015; Schwager *et al.* 2017).

Other dispersed paralogs that were present in clusters were the NK class families Nk7 and Tlx/C15 (fig. 2*A*). The *Pt-Nk7* paralogs are both expressed at very low levels throughout most of embryogenesis apart from S10 when they both increase in expression (fig. 3*A*). The *Pt-C15* paralogs, however, exhibit divergence in their timing and level of expression, with *Pt-C15b* showing increased expression around S7 to S10, compared with *Pt-C15a*, which is barely expressed at any of the ten stages (fig. 3*A*).

There were also several cases of dispersed (nonclustered) paralogs, which have diverged in the level and timing of their expression (fig. 3*A*). For example, *Pt-Hth2* is expressed throughout all ten stages, whereas *Pt-Hth1* is only expressed from S4 to S10 and these genes have demonstrably different expression patterning during limb development in this spider (Turetzek *et al.* 2017). Other dispersed paralogs that show aspects of divergence including *Pt-Gbx*, *Pt-Msx*, *Pt-Noto, Pt-Arx*, *Pt-Onecut*, *Pt-Hmbox*, and *Pt-Zfh* (fig. 3*A*), as well as the en/Inv family. *Pt-en* is expressed at S7 in the RNA-Seq data (fig. 3*A*), which is consistent with ISHs that show expression of *en* starts at early S8 in forming segments in *P. tepidariorum* (Schwager 2008). The *Pt-Inv1* paralog shows similar expression, however *Pt-Inv2* appears to be maternally loaded and down regulated at S2 when zygotic transcription starts (fig. 3*A*). Therefore, the timing of expression between *Pt-en*/*Pt-Inv* paralogs suggests that they have diverged in function.

A few dispersed paralogs exhibited very similar expression profiles such as *Pt-Pitx*, *Pt-Phox*, and *Pt-Vvl* (fig. 3*A*). However, it is possible that expression difference may occur later in development or during adult stages and this analysis does not account for any differences in the spatial expression pattern of these genes that may have occurred. This suggests that overall there has been evolutionary changes in the *cis*-regulation of most dispersed paralogs resulting in divergence in expression levels and transcriptional timing between paralogs.

### Divergence of Tandem Paralog Expression during *P. tepidariorum* Embryogenesis

Tandem duplicates, like dispersed duplicates, also exhibit both conserved and divergent expression profiles. The Emx family contains four paralogs, of which pairs of paralogs are found on two different scaffolds (fig. 2*A*). Paralogs *Pt-Emx1* and *Pt-Emx2* have similar expression, which increases from S6 to S10 (fig. 3*A*). In contrast the other two paralogs, *Pt-Emx3* and *Pt-Emx4*, are both expressed later from S7/S8 to S10 (fig. 3*A*). There is some early expression of *Pt-Emx4*, however, overall it appears that *Pt-Emx* paralogs that are on the same scaffold have more similar expression profiles.

The Irx family is also represented by four paralogs, two found in tandem (*Pt-Irx1* and *Pt-Irx2*) and two dispersed (*Pt-Irx3* and *Pt-Irx4*) (fig. 2*C*). The tandem duplicates are both expressed only at S10 (fig. 3*A*), while *Pt-Irx3* is expressed only at S3 and the *Pt-Irx4* paralog is expressed from S2 to S10 at fairly consistent levels (fig. 3*A*).

The Lim1/5 family is represented by two paralogs on one scaffold and a third paralog on a separate scaffold (fig. 2*D*). The two *Pt-Lim1/5* paralogs on the same scaffold had very similar expression, with low levels at S3 but stronger expression at S10 (fig. 3*A*). In contrast the single *Pt-Lim1/5* paralog on the other scaffold was expressed from S7 to S10 (fig. 3*A*).

The remaining tandem duplicates, *Pt-BarH*, *Pt-Prop*, and *Pt-Shox*, all showed divergent expression between paralogs (figs. 2*A* and *B* and 3*A*). For example, the *Pt-BarH1* paralog is strongly expressed from S1 to S6, whereas the other paralog appears to be expressed only in S1 and then again at S10 (fig. 3*A*).

### Comparison of Duplicated *P. tepidariorum* Homeobox Gene Expression with Single Copy Orthologs in *P. opilio*

To polarize the expression patterns of duplicated homeobox genes in a phylogenetic context, we analyzed the embryonic expression patterns of a subset of duplicated homeobox gene families in *P. tepidariorum* and compared the expression of selected spider genes to their single copy orthologs in *P. opilio*.

**Fig. 4.**
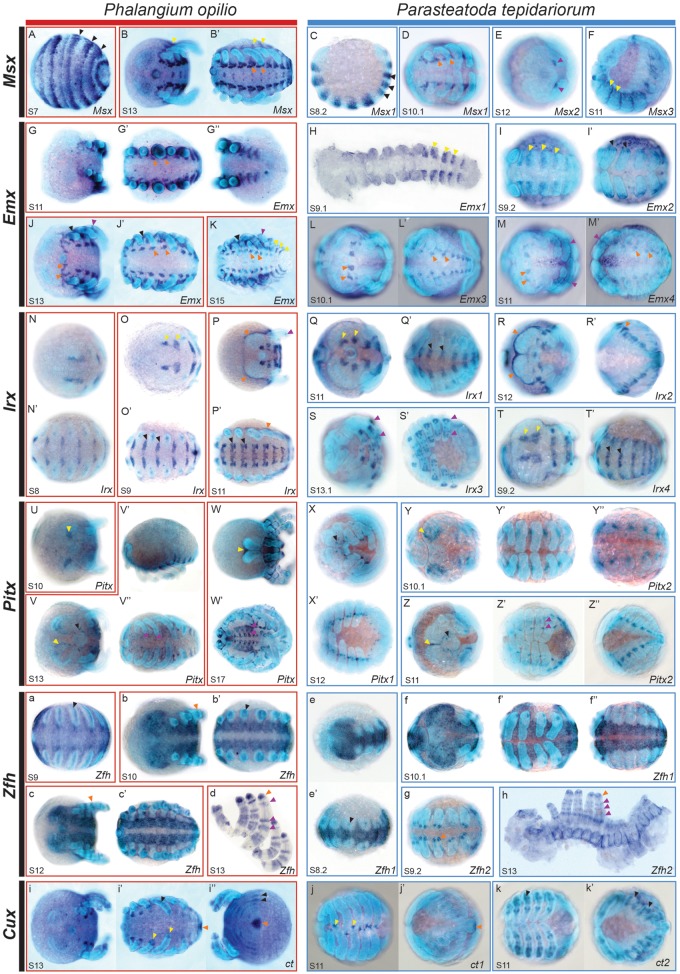
Expression of *Parasteatoda tepidariorum* paralogs compared with single copy orthologs in *Phalangium opilio*. Expression patterns of *Msx* (*A*–*F*), *Emx* (*G–M*′), *Irx* (*N–T*′), *Pitx* (*U–Z*″), *Zfh* (*a–h*), and *Cux* (*i–k*′) genes in *P. tepidariorum* (blue boxes) and *P. opilio* (red boxes). The early striped expression of *Po-Msx* (*A*) matches that of *Pt-Msx1* (*C*), indicated by black arrows. The patches of *Po-Msx* expression (*B*′) in each segment along the ventral midline are similar to *Pt-Msx1* (*D*), shown with orange arrows. Expression of *Po-Msx* and *Pt-Msx3* (*B*, *B*′ and *F*) are similar in the region around the base of the appendages (yellow arrows). *Pt-Msx2* has undergone possible neofunctionalization (*E*, purple arrows), with expression in the chelicerae that is not seen for *Po-Msx*. There is similar expression of *Po-Emx* (*K*) in the lateral parts of the opisthosoma compared with *Pt-Emx1* (*H*) and *Pt-Emx2* (*I*), shown with yellow arrows. The expression of *Po-Emx* around the base of the appendages is only seen for *Pt-Emx2* (*I*′), black arrows. The other two *P. tepidariorum* paralogs, *Pt-Emx3* and *Pt-Emx4*, both have expression in the precheliceral region and in patches along the ventral midline, which is also present in *P. opilio* (*G*′ and *J–M*′), indicated by orange arrows. The *Po-Emx* expression in the limbs (*J* and *K*) is similar to *Pt-Emx4* (*M* and *M*′), purple arrows. The expression of *Po-Irx* in the precheliceral region (*N*, *O*, and *P*) is seen for *Pt-Irx1* (*Q*) and *Pt-Irx4* (*T*), shown by yellow arrows. These two paralogs also have expression in the opisthosoma (*Q*′ and *T*′), which matches *Po-Irx* (*N*′, *O*′ and *P*′), black arrows. The expression of *Po-Irx* around the germ band (*P* and *P*′) can be seen for *Pt-Irx2* (*R* and *R*′), indicated by orange arrows. There is possibly more elaborate expression of *Pt-Irx3* (*S* and *S*′) in the limbs compared with *Po-Irx* (*P*), shown by purple arrows. *Pt-Pitx1* and *Pt-Pitx2* expression along the ventral midline (*X*′, *Y*′ and *Z*′), which in combination are similar to that seen for *Po-Pitx* (*V*″ and *W*′) (purple arrows). Expression of *Po-Pitx* in the precheliceral anterior furrows (*U*, *V*, and *W*, yellow arrows) is seen for *Pt-Pitx2* (*Y* and *Z*, yellow arrows). However the small dots of *Po-Pitx* expression around the stomodeum (*V*, black arrows) is shared between the two *Pt-Pitx* paralogs (*X* and *Z*, black arrows). Expression of *Po-Zfh* along the ventral midline (*a–c*′) is seen for the *Pt-Zfh1* paralog (*e–f*″). The later expression of *Po-Zfh* in the distal tips of limbs (*b* and *d*, orange arrows), in bands along the limbs (*d*, purple arrows) and faint expression throughout the embryo is mirrored by *Pt-Zfh2* (*g* and *h*). The expression of *Po-ct* (*i–i*″) has clearly subfunctionalized in *P. tepidariorum* with *Pt-ct1* having expression in distal tips of limbs (*j*, yellow arrows) and in the posterior of the germ band (*j*′, orange arrows). The expression of *Pt-ct2* (*k* and *k*′) resembles the striped expression of *Po-ct* in the opisthosoma (*i*″) and in the mesoderm of the appendages (*i*′), indicated by black arrows. All embryos are orientated with the anterior to the left. Images within a box are different views of the same embryo. Images *H* and *h* are flat mounted embryos. One side of the prosoma has been removed in (*h*) to aid flat mounting. Opisthosomal limbs have been dissected from *P. opilio* in (*d*).

The Msx family provides a likely example of neofunctionalization in the spider (fig. 4*A–F*). The likely ancestral expression pattern of this gene, possibly represented by *Po-Msx*, is mostly maintained in *Pt-Msx1* (fig. 4*A–D*). *Pt-Msx2* has probably gained a new expression domain in the chelicerae (fig. 4*E*). *Pt-Msx3* is also expressed in a conserved pattern at the base of the prosomal appendages (fig. 4*F*).

While we observed an apparent case of neofunctionalization in the Msx family there were several families (*Emx*, *Irx*, *Pitx*, *Zfh*, and *Cux*) that appear to have undergone subfunctionalization. In the Emx family, the expression pattern of the single copy of *Po-Emx* is subdivided between the four paralogs found in *P. tepidariorum* (fig. 4*G*–*M*′). Expression of the tandem paralogs *Pt-Emx1* and *Pt-Emx2* was observed in stripes in the anterior of each opisthosomal segment and *Pt-Emx2* also has expression at the base of prosomal appendages. In contrast, both *Pt-Emx3* and *Pt-Emx4* are expressed in the precheliceral segment and in patches in each segment along the ventral midline, which collectively form a similar expression seen for *Po-Emx* (fig. 4*G′ and J–M′*). Therefore, expression of *Pt-Emx* paralogs is most similar between the tandem paralogs (fig. 3*A*) consistent with the RNA-Seq profiles of these genes in *P. tepidariorum*. Nevertheless, some differences are still present between tandem duplicates, mostly in their prosomal appendage domain.

Another likely case of subfunctionalization occurs in the Irx family (fig. 4*N*–*T*′). In this family, *Pt-Irx1*, *Pt-Irx2*, and *Pt-Irx4* appear to have subdivided the expression pattern between them compared with *Po-Irx* (fig. 4*N*–*P*′). *Pt-Irx1* and *Pt-Irx4* have very similar expression domains, with expression in patches in the precheliceral segment and along the anterior boarder of prosomal and opisthosomal segments (fig. 4*Q*, *Q*′, *T*, and *T*′). However, *Pt-Irx4* expression extends more laterally in the opisthosomal segments, compared with *Pt-Irx1*. Furthermore, the onset of *Pt-Irx4* expression is earlier and continues until later in embryogenesis compared with *Pt-Irx1*. The other expression domain of *Po-Irx* around the dorsal boundary edge of the germ band is shared with the *Pt-Irx2* paralog. Finally, *Pt-Irx3* has possibly gained a completely new domain in the prosomal appendages of later stages and therefore possibly represents another case of neofunctionalization in *P. tepidariorum* (fig. 4*S*).

For the single copy of *Po-Pitx* (fig. 4*U*–*W*′), expression during embryogenesis was observed in the precheliceral region, which closely resembled the expression of *Pt-Pitx2* in precheliceral region of this spider (fig. 4*Y* and *Z*). Other expression domains of *Po-Pitx* around the stomodeum and the patches along the ventral midline were shared between the *Pt-Pitx* paralogs (fig. 4*X*–*Z*″), indicating possible subfunctionalization of *Pt-Pitx* paralogs.

The *Pt-Zfh* paralogs have also undergone expression divergence (fig. 4*e–h*) such that they represent subfunctionalization compared with *Po-Zfh* expression (fig. 4*a–d*). *Po-Zfh* expression initially starts along the ventral midline of the germ band with emerging expression within the opisthosomal appendages (fig. 4*a*–*b*′). This expression is mirrored by the *Pt-Zfh1* paralog (fig. 4*e*′), whereas the expression of *Pt-Zfh2* matches that of later *Po-Zfh* expression, with bands of expression in the limbs and faint expression surrounding the coxa and opisthosomal organs (fig. 4*d* and *h*).

*Po-ct* expression has also probably been subfunctionalized between *Pt-ct* paralogs (fig. 4*i–k*′). *Po-ct* is expressed in the tips of the prosomal appendages and at the very posterior of the germ band matching the expression of *Pt-ct1*, while the expression of *Po-ct* in the mesoderm of prosomal appendages, and opisthosoma matches *Pt-ct2* expression (fig. 4*i–k*′).

We also found that expression of *Hmx* paralogs in *P. tepidariorum* were highly divergent and again these paralogs perhaps represents an additional example of subfunctionalization (supplementary fig. 2*I–K*, Supplementary Material online). *Pt-Hmx1* is mainly expressed in the prosomal appendages while *Pt-Hmx2* is expressed in a pair of cell clusters in the precheliceral region (supplementary fig. 2*I–K*, Supplementary Material online).

Loss of embryonic expression was found in three of the ten families analyzed (Gbx, Dbx, and Vnd), where one paralog has retained the likely ancestral pattern as compared with *P. opilio*, while the expression of the other could not be detected during *P. tepidariorum* embryogenesis by ISH (supplementary fig. 2*A–H′*, Supplementary Material online). Additionally, in the case of *Pt-Gbx2*, only the prosomal appendage expression observed in *Po-Gbx* is conserved (supplementary fig. 2*C–E*, Supplementary Material online), while this gene has also possibly gained a novel expression domain in the opisthosomal limb buds (supplementary fig. 2*F*, Supplementary Material online). It remains possible that the paralogs for which we did not detect expression during embryogenesis are expressed later during juvenile or adult stages.

## Discussion

### Homeobox Gene Repertoires in Chelicerates

Homeobox genes encode an important group of transcription factors that regulate a wide range of developmental processes (Zagozewski *et al.* 2014; Bataille *et al.* 2015; Du and Taylor 2015; Zuniga 2015; Krumlauf 2016). Consequently they have received substantial attention and are often characterized and compared within and between animal genomes to better understand their evolution and development. Among arthropods, the insects have been sampled the most extensively and robustly, but there has been limited characterization of these genes in other arthropod groups. For example, among the chelicerates, systematic analysis of the homeobox gene repertoires has only been carried out previously for horseshoe crabs and the scorpion *M. martensii* (Di *et al.* 2015; Kenny *et al.* 2015). Therefore, in order to better understand the homeobox repertoires in chelicerates, we surveyed the two spiders *P. tepidariorum* and *P. phalangioides*; another scorpion, *C. sculpturatus*; the pseudoscorpions *N. carcinoides, Hesperochernes* sp.; the harvestman *P. opilio*, and the tick *I. scapularis*.

Overall we found a similar complement of homeobox classes and families verifying that chelicerates share and have retained similar homeobox repertoires to other arthropods (fig. 1). However several families were observed that are possibly specific to scorpions (Nk8 and Six7), and the Nedx family in spiders was not found in other arachnids except one of the pseudoscorpions. These particular families may therefore regulate lineage specific features during scorpion and spider development. Furthermore, the Barx family, which is found in chelicerates but not in other arthropods, may coordinate specific aspects of chelicerate development.

Aside from the incomplete data set from the pseudoscorpions and the harvestman, we found the fewest homeobox families in the tick *I. scapularis* indicating that they have either been lost in this arachnid or there is incomplete sequence information for all families. However, the lineage of parasitiforms, and their putative sister group, the acariforms, also exhibit a greater loss of conserved miRNA families compared with other arachnid lineages (Leite *et al.* 2016). Mite genomes in particular can exhibit marked genome compaction, dynamic rearrangements of homeobox clusters, and associated loss of many transcription factors (Hoy 2009; Grbic *et al.* 2011). Therefore it is likely that there is actual loss of homeobox genes in *I. scapularis*. Interestingly, we also observed long-branch lengths for several tick homeodomains, but it is not known if these functional changes are related to the loss of genes, to rapid evolution of gene function, or to the underlying accelerated rate of evolution inherent to this order (Sharma, Schwager, et al. 2014). Note that while we found only a few families in the two pseudoscorpion species, this likely reflects their representation in the transcriptomes analyzed rather than true losses in this lineage.

### Expansion of Homeobox Genes after WGD in the Ancestor of Arachnopulmonates

Previous work identified duplicated homeobox genes in chelicerates (Nossa *et al.* 2014; Di *et al.* 2015; Kenny *et al.* 2015), such as Hox genes in spiders and scorpions (Schwager *et al.* 2007; Sharma, Schwager, *et al.* 2014; Sharma et al. 2015), as well as other homeobox genes involved in spider eye development (Samadi *et al.* 2015; Schomburg *et al.* 2015). However, apart from a scorpion and horseshoe crabs there was no previous systematic analysis of homeobox duplication in chelicerates and in particular how these repertoires have been shaped by WGD in the ancestor of arachnopulmonates.

We found many more duplicated homeobox families in arachnopulmonate species (51–59%) compared with other arthropods surveyed, including *I. scapularis* (24%), *P. opilio* (3%), pseudoscorpions (19% and 6%), and several mandibulates (19%) (fig. 1). Indeed, the proportion of duplicated homeobox families found in *P. tepidariorum* or *C. sculpturatus* is greater than found in either the BUSCO (41%) or OMA (20.5%) data sets (Schwager *et al.* 2017). In fact 18 homeobox families were represented by two paralogs in all four arachnopulmonates but were only single copy in all other arthropods surveyed. This makes up a considerable proportion of the 63–78 duplicates identified in *P. tepidariorum* and *C. sculpturatus* compared with mandibulates and ticks with respect to the BUSCO-Ar database.

It was previously shown that two clusters of Hox genes have been retained in arachnopulmonates following WGD, whereas only one Hox cluster with single copies of most Hox genes is found in *P. opilio*, *I. scapularis*, and *T. urticae* (Sharma *et al.* 2012; Pace *et al.* 2016). Indeed this appears to be a general consequence of WGD: there are two complete and two partial clusters of Hox genes in horseshoe crabs (Nossa *et al.* 2014). In addition, in vertebrate lineages multiple clusters of Hox genes have been produced by several WGD events (Hoegg and Meyer 2005; Mungpakdee *et al.* 2008; Pascual-Anaya *et al.* 2012).

We also found evidence for the duplication of clusters of other homeobox genes in arachnopulmonates in the form of duplicated ANTP (NK cluster), SINE, TALE, and LIM class genes (fig. 2*A*, *C*, and *D*). The inferred ancestral order of arachnopulmonate NK cluster genes (*Nk7*, *Lbx*, *Tlx*, *bap*, *tin*, *Msx*) is consistent with their predicted order in the protostome–deuterostome ancestor (Garcia-Fernandez 2005; Ferrier 2016), requiring just an inversion containing *Lbx* and *Tlx* (fig. 2*A*). Other ANTP class genes in *P. tepidariorum* are also clustered, which is suggestive of remnants of the mega cluster, however these were not retained as duplicates (fig. 2). A HRO cluster containing *Hbn, Rax2*, and *Otp* was also present, and provides further evidence, along with data from *S. maritima*, that this cluster is a feature of arthropods and other protostomes (fig. 2*B*) (Mazza *et al.* 2010; Chipman *et al.* 2014; Ferrier 2016). However, the order of the three genes in *P. tepidariorum* is different to other arthropods, suggesting that there has been an inversion in the lineage leading to this spider (Mazza *et al.* 2010).

In insects and myriapods, the SINE/Six cluster has degraded and all three genes are dispersed in the genome (Chipman *et al.* 2014; Ferrier 2016). This suggests that the SINE/Six cluster was present in the arthropod ancestor and then has subsequently been degraded in mandibulates but retained in chelicerates. The clusters of ANTP, PRD, SINE, TALE, and LIM class genes in *P. tepidariorum* suggests that spiders have retained many features of the hypothetical clustering of homeobox genes in the bilaterian ancestor (Ferrier 2016). Furthermore, several of these clusters are duplicated and there are different patterns of gene loss/retention and rearrangements, for example, fewer genes have been lost in the Hox cluster compared with the NK cluster.

Retention of gene duplicates in arachnopulmonates has also been observed for other important developmental genes including Wnts and *frizzled4*, and *dachshund* (*dac*), as well as venom and silk genes (Schwager *et al.* 2007, 2017; Janssen *et al.* 2010, 2015; Haney *et al.* 2014, 2016; Clarke *et al.* 2015; Pechmann *et al.* 2015; Samadi *et al.* 2015; Schomburg *et al.* 2015; Turetzek *et al.* 2016, 2017). Furthermore, miRNAs are also pervasively duplicated in arachnopulmonate genomes (Leite *et al.* 2016). This suggests that the retention of duplicated homeobox genes and other developmental toolbox genes after WGD in arachnopulmonates has played an important role in the evolution of development of these animals. The high rate of retention of duplicated homeobox genes after WGD in arachnopulmonates is similar to that observed after the two rounds of WGD in vertebrates (Dehal and Boore 2005; Maere *et al.* 2005; Holland *et al.* 2008; McGrath *et al.* 2014; Schwager *et al.* 2017). Indeed most of the homeobox gene families duplicated in arachnopulmonates are also duplicated in vertebrates, but interestingly the Noto, Drgx, Hmbox families are only duplicated in the former (supplementary fig. 3, Supplementary Material online) (Zhong *et al.* 2008; Zhong and Holland 2011). This indicates that arachnopulmonates and vertebrates have independently retained and utilized duplicated copies of these important transcription factors and this likely contributed to the developmental evolution, novel phenotypes, and adaptation of these two phyla. Furthermore, families that were only present as single copies in vertebrates and arachnopulmonates were Bsx, Hlx, and Mkx, which indicates that these families fail to retain duplicates in both lineages after WGDs. An intriguing counterpoint for future investigation is therefore horseshoe crabs, which have been shown to have likely undergone two rounds of WGD, but exemplify morphological external stasis and evolutionary relictualism (Sharma, Schwager, *et al.* 2014; Kenny *et al.* 2015; Schwager *et al.* 2017).

### Divergence in the Expression of Homeobox Paralogs

How has the ancestral WGD in arachnopulmonates contributed to their evolution and the development of lineage specific features? It has already been shown that one paralog of *dac* in the spider has a distinct and novel role (by comparison to the ancestral function of this gene within Arthropoda), being responsible for patterning the distal boundary of the arachnid-specific podomere, the patella (Turetzek *et al.* 2016). Furthermore, the arrangement of structures in the opisthosoma of scorpions coincides with the staggered expression of paralogous Hox gene expression (Sharma, Schwager, *et al.* 2014), suggesting that divergences in Hox paralogs may in part be responsible for innovations of the scorpion body. Moreover, the Hox paralogs of spiders have also divergences in their temporal and spatial expression (Schwager *et al.* 2007, 2017), while other homeobox paralogs also show differential expression among the developing eyes (Samadi *et al.* 2015; Schomburg *et al.* 2015).

In our study we did not identify any homeobox gene paralogs in *P. tepidariorum* with the same temporal expression profile (fig. 3*A*), and ISHs on a subset of paralogs also showed divergence in the spatial expression between *P. tepidariorum* paralogs. Comparisons of gene expression of *P. tepidariorum* paralogs with their single copy ortholog in *P. opilio* suggest that most of the surveyed paralogs have likely undergone subfunctionalization, usually in the developing appendages and nervous system. The *P. tepidariorum Msx* genes have also apparently been subject to possible neofunctionalization in the case of *Msx2* in developing chelicerae.

The expression of *Pitx* and *Zfh* genes between *P. opilio* and *P. tepidariorum*, in conjunction with their known expression in *Drosophila*, provide particularly strong evidence for subfunctionalization. In *Drosophila*, *Pitx* is expressed in several tissues including a subset of ventral somatic muscles and in neural cells (Vorbrüggen *et al.* 1997). These expression patterns in *Drosophila* are consistent with *Po-Pitx* expression (fig. 4), suggesting that this expression pattern is ancestral. In *P. tepidariorum*, both *Pitx* paralogs also show metameric patterning along the ventral neuroectoderm, with *Pt-Pitx1* most similar to the *Drosophila* and *P. opilio* CNS expression and *Pt-Pitx2* showing both CNS and mesodermal expression (fig. 4*U*–*Z*″).

The expression patterns of *Zfh* in *Drosophila* and *P. opilio* are also very similar, again implying the ancestral expression of this gene (fig. 4*a–d*). Early expression of the *Drosophila Zfh2* ortholog is seen in the brain and ventral CNS (Lai *et al.* 1991), with later expression seen in leg imaginal discs as an initially broad domain at the centre of the disc that develops into rings of expression in each leg segment and in a domain throughout the tarsus (Guarner *et al.* 2014). These patterns are similar to that seen for *P. opilio* (fig. 4*a–d*). However in *P. tepidariorum*, early CNS expression and later limb expression has been divided between the *Zfh* paralogs. *Pt-Zfh1* is strongly expressed in the CNS and initial limb buds, while *Pt-Zfh2* is observed in the later pattern expression in rings and at the distal tips of the limbs (fig. 4*e–h*). This expression divergence observed for the *Pitx* and *Zfh* paralogs exemplify the pervasive temporal and spatial subfunctionization of genes that has likely occurred in the spider and probably other arachnopulmonates.

## Conclusion

Our study has revealed the first comparative genomic picture of the repertoires of homeobox genes in arachnids. This shows that there has been a high level of gene retention of these developmental genes since the WGD in the common ancestor of arachnopulmonates. Furthermore, most of the *P. tepidariorum* homeobox gene paralogs exhibit differences in their timing and spatial expression, and when compared with their single copy homologues in *P. opilio*. This suggests there has been pervasive subfunctionalization and/or neofunctionalization of these genes since WGD. It will be interesting to further investigate the roles of these genes in spider development to ascertain their contribution to the evolution of development and diversification of these arachnids, especially with respect to emergence of novel traits including silk glands and book lungs. Furthermore, future comparisons of ohnologs between arachnopulmonates and vertebrates should provide exciting new insights into the general consequences of WGD in animals.

## Materials and Methods

### Identification of Homeobox Genes in Arachnids

To identify homeobox genes in arachnid species, we analyzed both existing resources and also new transcriptomic data generated in this study. Existing protein predictions were collected for the tick *Ixodes scapularis* (PRJNA16232), the harvestman *Phalangium opilio* (PRJNA236471), and the pseudoscorpion *Hesperochernes* sp. (PRJNA254752).

For further characterization of homeobox genes in arachnids we also generated de novo transcriptomes for the spider *Pholcus phalangioides* and the pseudoscorpion *Neobisium carcinoides*. For *P. phalangioides* RNA isolation, library preparation and sequencing with Illumina HiSeq2000 was previously described (Janssen *et al.* 2015). A de novo transcriptome assembly (Turetzek N, Torres-Oliva M, Kaufholz F, Prpic NM, Posnien N, in preparation) was performed with Trinity version r20140717 (Haas *et al.* 2013) with the following settings: –seqType fq –JM 240 G – run_as_paired –CPU 6 and using Trimmomatic for quality trimming and filtering (Bolger *et al.* 2014). For the pseudoscorpion *N. carcinoides*, RNA was extracted from the whole body, sequenced with Illumina HiSeqll and de novo assembly of the transcriptome was carried out using Trinity v 2.0.3 (Grabherr *et al.* 2011) under default parameters and using Trimmomatic for quality control. The raw sequence reads for *P. phalangioides* and the pseudoscorpion *N. carcinoides* have been deposited in the SRA with accession numbers PRJNA448805 and PRJNA438779, respectively.

Longest open reading frames (ORFs) were predicted from the transcriptomes of *P. phalangioides* and the pseudoscorpion *N. carcinoides* as well as from the existing nucleotide transcriptome of the harvestman *P. opilio* (PRJNA236471) and the pseudoscorpion *Hesperochernes* sp. (PRJNA254752) using TransDecoder v3.0.0 (Haas *et al.* 2013). To retain putative proteins the sequence homology and protein domains of predicted ORFs were then analyzed, respectively, with BLASTP v2.2.28+ (e-value 1e^−6^) (Altschul *et al.* 1990) using the UniProt Swiss-Prot database (UniProt 2015), and HMMER v3.1 (Wheeler and Eddy 2013) using the Pfam v30.0 database (Finn *et al.* 2016).

The protein sequences from *P. phalangioides*, *I. scapularis*, *P. opilio*, and the two pseudoscorpions were then searched for the presence of homeodomain sequences using BLASTP v2.2.28+ (Altschul *et al.* 1990) with query amino acid homeodomain sequences from all ten species in HomeoDB (Zhong *et al.* 2008; Zhong and Holland 2011) combined with homeodomain sequences from *P. tepidariorum* (Schwager *et al.* 2017), *C. sculpturatus* (Schwager *et al.* 2017), *M. martensii* (Di *et al.* 2015), *Strigamia maritima* (Chipman *et al.* 2014). All the initial BLASTP hits with >30% percentage identity were retained. Next, the full protein sequences of the BLASTP hits were then analyzed using the Conserved Domain Database (CDD) search tool (Marchler-Bauer *et al.* 2015) to confirm the presence of homeodomains as well as annotate other functional domains. BLASTP hits that did not have homeodomains identified by CDD were removed. Transcripts within a species that had identical protein sequences predicted to encode homeodomains were manually checked and identical nucleotide transcripts or isoforms were removed. Specific BLAST searches for PROS class genes also identified a *Pros* gene (MMa30254) in *M. martensii* not reported previously by Di *et al.* (2015). All identified homeobox genes and their sequences are given in supplementary file 1, Supplementary Material online. By concentrating on the detection of homeobox genes based on the presence of homeodomains some partial transcripts of homeobox genes that lack this domain, or may have highly divergent homeodomains, may be missing in our data set.

### Phylogenetic Analysis of Arachnid Homeodomains

The predicted homeobox genes were then classified based on phylogenetic analysis of the homeodomain sequences they encode. Amino acid sequences of homeodomains from two spiders (*P. tepidariorum* and *P. phalangioides*), two scorpions (*C. sculpturatus* and *M. martensii*) two pseudoscorpions (*Hesperochernes* sp. and *N. carcinoides*), the harvestman *P. opilio*, the tick *I. scapularis*, the myriapod (centipede) *S. maritima* and three insects *Apis mellifera*, *Tribolium castaneum*, and *Drosophila melanogaster* were aligned with ClustalW (Larkin *et al.* 2007), excluding unusual PROS HPD sequences and the *Cs-Emx1* homeodomain because it has a large insertion.

Phylogenetic analyses, using only unique homeodomain sequence alignments, were performed in RAxML, with support levels estimated using the rapid bootstrap algorithm (1000 replicates) (Stamatakis *et al.* 2008), under the PROTGAMMALG model of amino acid substitution—that was identified as best fitting using a custom Perl script from the Exelixis Lab website (https://sco.h-its.org/exelixis/web/software/raxml/hands_on.html). Homeodomain proteins were classified based on the homology of their homeodomains to known homeodomain containing proteins and annotated with nomenclature following that of Holland *et al.* (2007).

### Synteny Analysis of Homeobox Genes in *P. tepidariorum*

To investigate the arrangement of homeobox genes in *P. tepidariorum* we used the high quality HiRise/DoveTail genome assembly (Schwager *et al.* 2017). The scaffold location and coordinates of the previously identified homeobox genes (Schwager *et al.* 2017) were extracted from the GFF file, which contains coordinates of AUGUSTUS gene models relative to the HiRise/DoveTail genome, and were used to calculate the gaps between genes.

### Analysis of Homeobox Gene Expression in *P. tepidariorum* Embryogenesis

Homeobox gene expression levels were analyzed during *P. tepidariorum* embryogenesis using RNA sequencing. RNA was extracted using the Dynabeads mRNA DIRECT Kit (Ambion) from 10–100 embryos of each successive developmental stage (stage [S]1–S4, S5 early and S5 late, S6–S8 and S10; Akiyama-Oda and Oda 2003; Mittmann and Wolff 2012). Two replicate sets of mRNAs were independently obtained from two pairs of parents. The mRNAs were fragmented using the NEBNext RNase III RNA Fragmentation Module (New England BioLabs) and then used to construct DNA libraries with the NEBNext Ultra Directional RNA Library Prep Kit for Illumina (New England BioLabs) and NEBNext Multiplex Oligos for Illumina (Index Primers Set 1, New England Biolabs). The libraries were sequenced using the 150-cycle format of the Illumina MiSeq Reagent Kit v3. The resulting sequence reads were subjected to adaptor trimming using the CLC Genomics Workbench 7.0.3 (Qiagen), and quality of the sequences was confirmed with FastQC v0.11.2 (Andrews 2011). The trimmed raw reads have been deposited in the SRA with accession number PRJNA448775 (Iwasaki-Yokozawa *et al.* 2018). Replicates for each stage were aligned to the *P. tepidariorum* reference transcriptome (Schwager *et al.* 2017) using TopHat v2 (Kim *et al.* 2013). Outputs files were sorted and indexed with Samtools v1.2 (Li *et al.* 2009) and RPKM expression levels were quantified using HTSeq-count (Anders *et al.* 2015) and custom Perl scripts. Heatmaps were generated in R v3.2.3 (R Core Team 2015) using the ComplexHeatmap package (Gu *et al.* 2016).

### *Parasteatoda tepidariorum* and *P. opilio* Cultures

An inbred culture of *P. tepidariorum* (from a strain collected in Göttingen, Germany) was maintained at Oxford Brookes University and fed on a diet of *Drosophila vestigial* mutants and *Gryllodes sigillatus*, with a 12:12 h light:dark cycle at 25°C. The culture of *P. opilio* was maintained at the University of Wisconsin, Madison, WI and fed on a diet of fish flakes supplemented with *Acheta domesticus* nymphs, with a 14: 10 light: dark cycle at 20°C.

### Cloning of Gene Fragments and Probe Synthesis

cDNA was generated using QuantiTech (Qiagen) with RNA extracted (Qiazol) from S1 to S14 *P. tepidariorum* embryos and from S7 to S17 for *P. opilio*. Gene fragments were amplified by PCR and cloned into the TOPO-TA vector (ThermoFisher Scientific). Primer sequences are provided in supplementary table 1, Supplementary Material online. RNA probes were transcribed with T3 (11031163001—Roche) or T7 polymerase (10881775001—Roche), with DIG RNA labeling mix (11277073910—Roche), from PCR fragments generated from TOPO-TA clones following standard protocols.

### In Situ Hybridization in *P. tepidariorum* and *P. opilio*

Colourmetric in situ hybridization (ISH) for *P. tepidariorum* and *P. opilio* was performed as previously described (Akiyama-Oda and Oda 2003; Sharma *et al.* 2012). Embryos were counterstained with DAPI (Roche—10236276001) for ∼20 mins to visualize nuclei. Embryos were imaged using a Zeiss Axio Zoom V.16 and a Nikon SMZ25, and overlays were generated in Photoshop CS6.

## Supplementary Material

Supplementary Data
