# Supplementary material for: Homeobox Gene Duplication and Divergence in Arachnids
Source: Mol Biol Evol. 2018 Jun 19;35(9):2240–53. doi: 10.1093/molbev/msy125 (PMC6107062; doi:10.1093/molbev/msy125)
Supplement: Supplementary Data [file msy125_supp.zip › SupTable1.docx]

## Supplementary Table 1: Primers for PCR amplification

| **Species** | **Gene** | **Forward primer** | **Reverse primer** | **Fragment size (bp)** |
| --- | --- | --- | --- | --- |
| Parasteatoda | Cux1 | GTTGGGCCTATCTATCCGCACT | CCACTGCTTGATGGAGGTGGTA | 756 |
| Parasteatoda | Cux2 | GGAGCAGAAGGAAGCCTTGAGA | TCCTGTTCGTTACCGCTGTCAT | 774 |
| Parasteatoda | Dbx1 | ACAGTTACGCCTTTTCTGGT | ACAGCCCTCCTCATCATTCC | 322 |
| Parasteatoda | Dbx2 | GCAAGTCCACATGTTACCCA | ACGATGTCACTCAAATCAGGC | 422 |
| Parasteatoda | Emx1 | TGCTTCTCCGGTTTCTGTCA | CGCACTTTTCATCACTGGTG | 737 |
| Parasteatoda | Emx2 | TTCCAAAACCAACACGACCT | GGTGACTGTGAACGGATGTA | 832 |
| Parasteatoda | Emx3 | CAGCCGAAGACCACATGAAC | GGTAGTGCCGTCATTTCTGG | 808 |
| Parasteatoda | Emx4 | CACCAACAAGAGCACCAGAA | GACGCACGACACAAATGAAT | 868 |
| Parasteatoda | Gbx1 | ACGAACAACCTTTACTAGCG | CCAAGCTGATTATGAACTGC | 240 |
| Parasteatoda | Gbx2 | GGGACTTTATTCAACCGGAGC | CAGAGCATCGGAAAGGGAGA | 651 |
| Parasteatoda | Hmx1 | ACCTCCCGCATTTTTACCGGTA | ACTAGGTAGAGGGGCACGTGTA | 709 |
| Parasteatoda | Hmx2 | GCACCTTCGATAGCCACAAG | AGCTTCTATGTCAGCTGCCA | 703 |
| Parasteatoda | Irx1 | CACCACAAAGTAGCCAGCAG | TCCCAAGTGAAAACAGCGTG | 920 |
| Parasteatoda | Irx2 | CGTAGAACAGCATCGTGTGA | TTTATTGCAGGGATGTCGCC | 862 |
| Parasteatoda | Irx3 | GCTCGCCATCATCACCAAAA | GAGAACCTCTTGACAGCACT | 826 |
| Parasteatoda | Irx4 | AATGCCAGGAAAGTGACTCC | TGTTAGCTCTCACACCGACT | 835 |
| Parasteatoda | Msx1 | GCAAGCCTCGAACTCCTTTC | CCGTTTGGCACCATCTATGA | 993 |
| Parasteatoda | Msx2 | GCGTGGATATGGAAGAGTCG | GGTGCCATCAGAGGTCCTAA | 556 |
| Parasteatoda | Msx3 | GAAGTCCAAGTCCTGCGTTA | TCGGTAAAGACACAATGGGG | 756 |
| Parasteatoda | Pitx1 | GACCCATCACAGACTGGACA | CCAGCATATGTCCCAAAGCC | 804 |
| Parasteatoda | Pitx2 | ACTTGCCTGACTCAGCATCT | TAAGGACAAGGAGGTGCAGG | 629 |
| Parasteatoda | Vnd1 | TCCAACAGACGCAACAACAA | TTGTTCTACCACCAACGCTG | 657 |
| Parasteatoda | Vnd2 | ACCTTTTGCACACATGGGTC | CTCTCTTTCTGGGGCTGACA | 505 |
| Parasteatoda | Zfh1 | CCAGAGACTACCCCAACACCTG | TAGGCAATGGCGTAACACCAGA | 568 |
| Parasteatoda | Zfh2 | AAGTTCCTTTGAGCATCGCAGC | GTCTCGGTCGAGGAAATGGACA | 501 |
| Phalangium | Cux | CGCCTTCCAGTTCTTCATCG | GGGGTAGGGGTCCATTGAAA | 686 |
| Phalangium | Dbx | TTGCAGGTCAAAATATGGTTCC | GGAGGGTTGCTATTGACTCC | 158 |
| Phalangium | Emx | GTTAGCCGTACCCGTTTCAC | TGGAGTATGAAACCGTGGCT | 648 |
| Phalangium | Gbx | GTTCTTCGCCGGCTTCTTTC | TTTTCGGAGCGTTAGGGTTC | 393 |
| Phalangium | Irx1 | AAATTACCAACGCCTAGCCG | CATCGTCGCACCTGTTTCTG | 512 |
| Phalangium | Msx | CCAAATTCACCCTCACCGAC | GTTTCCGGTTGCTCTTGTGT | 829 |
| Phalangium | Vnd | CCTACATCCCTCCAACAGCA | CCGAACCACCACCGTCTATA | 783 |
| Phalangium | Pitx | GAGATTCAACGTCGTCAGGC | AGGTGGTGTGGGATTGCTTA | 572 |
| Phalangium | Zfh | GTCTGAACGCCTCGATGAAC | GAATTTCGGGTCTTGCGGTT | 983 |
