# Supplementary figures and images for: Homeobox Gene Duplication and Divergence in Arachnids
Source: Mol Biol Evol. 2018 Jun 19;35(9):2240–53. doi: 10.1093/molbev/msy125 (PMC6107062; doi:10.1093/molbev/msy125)

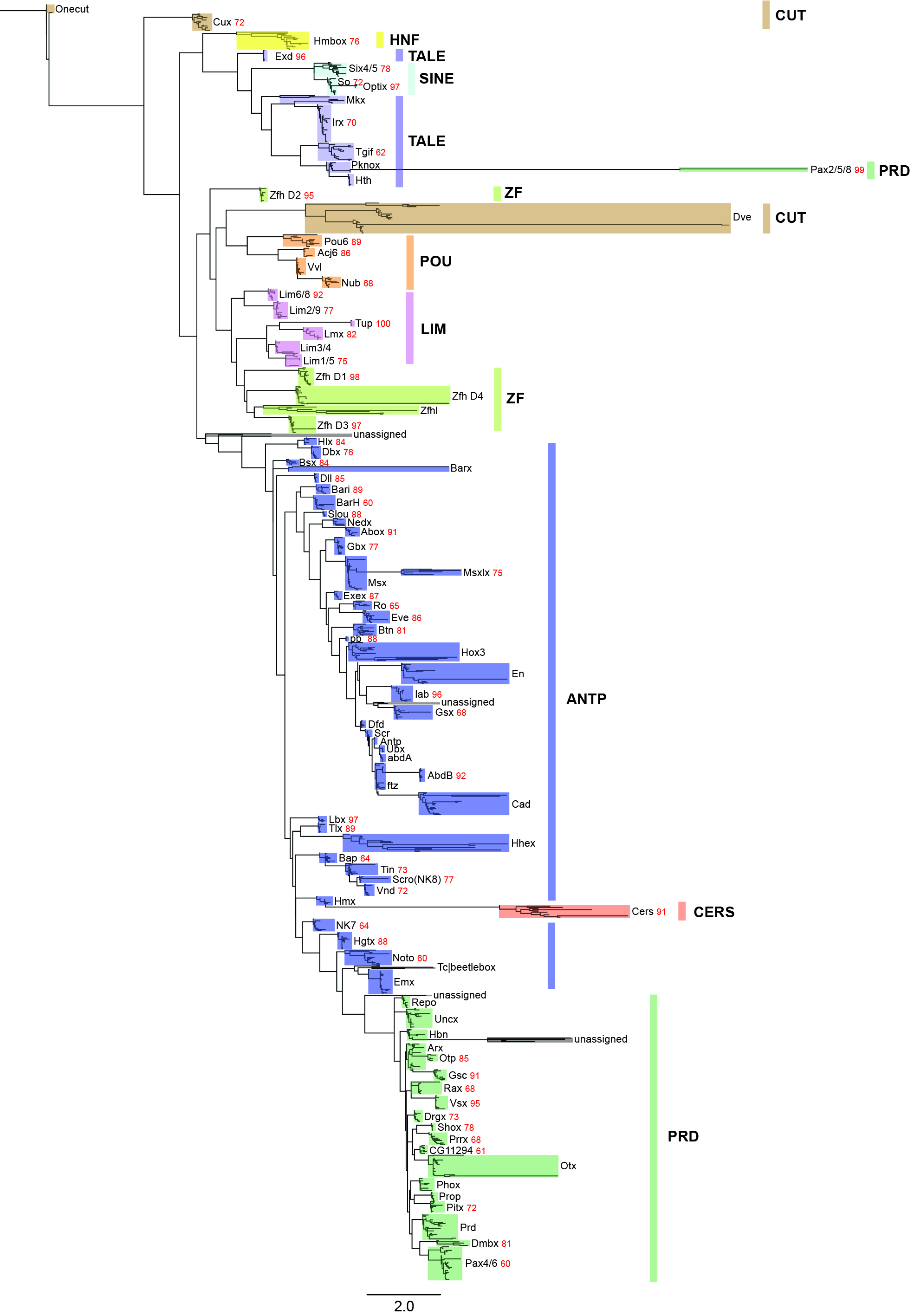

Supplement: Supplementary Data [file msy125_supp.zip › SupFig1.jpg]

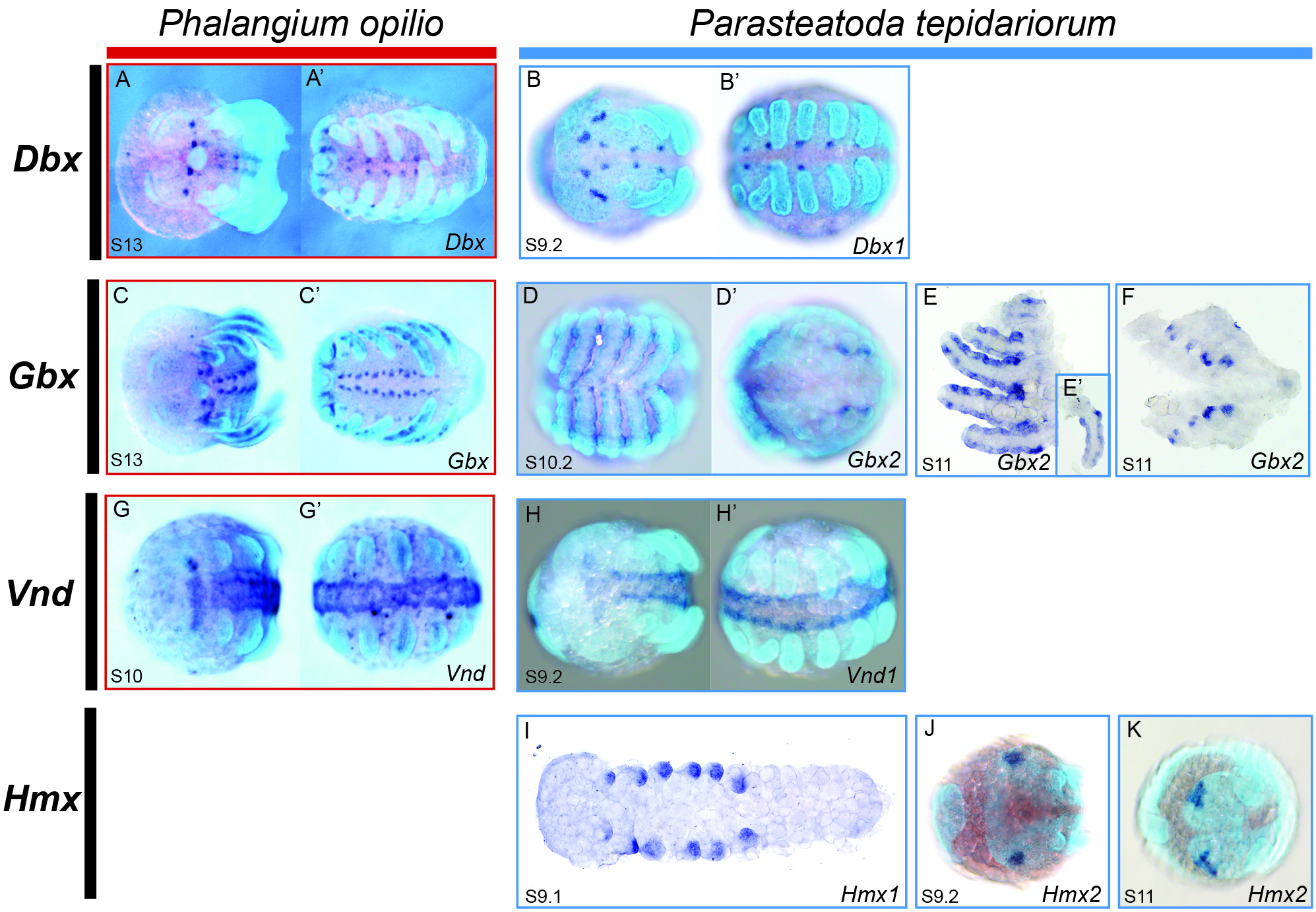

Supplement: Supplementary Data [file msy125_supp.zip › SupFig2.jpg]

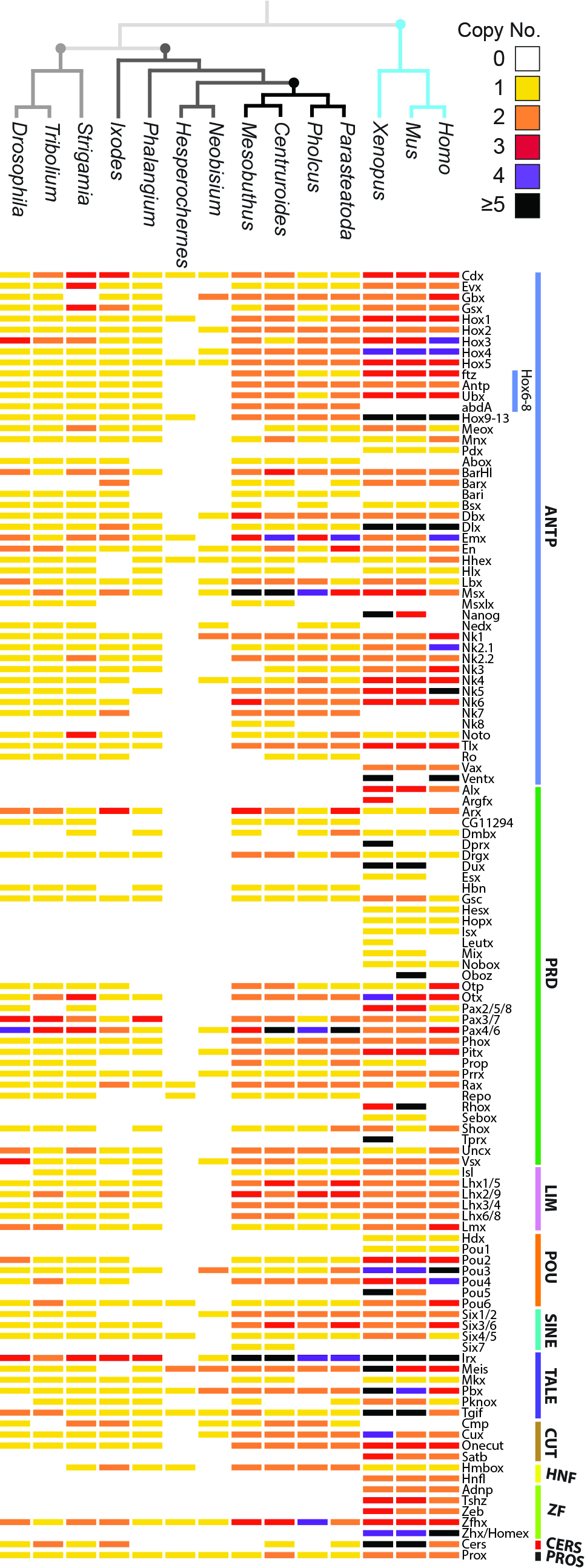

Supplement: Supplementary Data [file msy125_supp.zip › SupFig3.jpg]
